# Merkel cells and keratinocytes in oral mucosa are activated by mechanical stimulation

**DOI:** 10.14814/phy2.15826

**Published:** 2024-01-21

**Authors:** Chi‐Kun Tong, Yalda Moayedi, Ellen A. Lumpkin

**Affiliations:** ^1^ Department of Physiology and Cellular Biophysics Columbia University Medical Center New York New York USA; ^2^ Department of Dermatology Columbia University Medical Center New York New York USA; ^3^ Present address: Departments of Neurology and Otolaryngology‐Head and Neck Surgery Columbia University New York NY USA; ^4^ Present address: Department of Molecular and Cell Biology Helen Wills Neuroscience Institute, University of California, Berkeley Berkeley CA USA

**Keywords:** calcium imaging, Merkel cells, oral mucosa, PIEZO2

## Abstract

The detection of mechanical qualities of foodstuffs is essential for nutrient acquisition, evaluation of food freshness, and bolus formation during mastication. However, the mechanisms through which mechanosensitive cells in the oral cavity transmit mechanical information from the periphery to the brain are not well defined. We hypothesized Merkel cells, which are epithelial mechanoreceptors and important for pressure and texture sensing in the skin, can be mechanically activated in the oral cavity. Using live‐cell calcium imaging, we recorded Merkel cell activity in ex vivo gingival and palatal preparations from mice in response to mechanical stimulation. Merkel cells responded with distinct temporal patterns and activation thresholds in a region‐specific manner, with Merkel cells in the hard palate having a higher mean activation threshold than those in the gingiva. Unexpectedly, we found that oral keratinocytes were also activated by mechanical stimulation, even in the absence of Merkel cells. This indicates that mechanical stimulation of oral mucosa independently activates at least two subpopulations of epithelial cells. Finally, we found that oral Merkel cells contribute to preference for consuming oily emulsion. To our knowledge, these data represent the first functional study of Merkel‐cell physiology and its role in flavor detection in the oral cavity.

## Graphical abstract

This article explores the potential mechanism of food texture detection in the oral cavity, focusing on Merkel cells' activation in response to mechanical stimulation. The study reveals region‐specific differences in Merkel cell activation thresholds and unexpected activation of oral keratinocytes. In our behavioral study, oral Merkel cells are found to contribute to the preference for consumption of an oily emulsion, shedding light on their role in flavor detection.

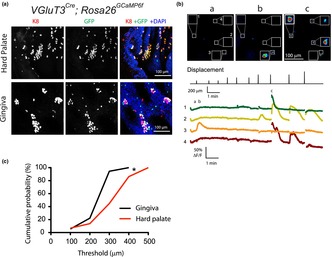

## 1 INTRODUCTION

Flavor is a multimodal sensory experience influenced by food texture, taste, smell, sight, and other factors (Small, 2012). Food and drink textures, such as hardness, crispness, crunchiness, springiness, and viscosity, reflect their mechanical properties and could affect consumers' perception of the quality. Food of the same taste but different textures produces different masticatory behaviors (Le Révérend et al., 2016), and influences energy intake as well as metabolism in rats and humans (Bae et al., 2014; Forde et al., 2013; Oka et al., 2003). How food texture features are transduced in the oral cavity, transmitted from the peripheral to the central nervous system, and how these signals are integrated with other sensory systems to shape flavor is poorly understood.

In *Drosophila melanogaster*, groups of mechanosensitive neurons and mechanically activated channels have been identified to play critical roles in the detection of food texture, smoothness, and size (Jeong et al., 2016; Li & Montell, 2021; Sanchez‐Alcaniz et al., 2017; Zhang et al., 2016); however, the functions of mechanoreceptive structures and their respective roles for distinguishing food textures in mammals are largely unknown. Recent efforts have begun to unravel the complexity of mechanosensitive afferents innervating the mouse and human tongue (Grayson et al., 2019; Moayedi et al., 2018, 2021; Trulsson & Essick, 1997, 2010; Yokota & Bradley, 2017). In particular, mechanosensitive GDNF‐Ret+ neurons in geniculate ganglion have been identified that responded only to light stroking stimuli in tongue (Donnelly et al., 2018; Yokota & Bradley, 2017), indicating that genetically distinct primary afferents terminating in the tongue are responsible for mechanotransduction. However, little is known about the mechanosensitivity of other oral surfaces, such as gingival and hard palatal epithelia, and whether specialized cells play a role in initializing mechanosensitive responses.

In skin, epithelial Merkel cells are mechanosensory cells that boost mechanosensory afferent firing rates during dynamic stimuli and transduce sustained pressure (Maksimovic et al., 2014). In the gastrointestinal (GI) tract, specialized sensory enteroendocrine cells (EECs) interact intimately with luminal contents, and a subpopulation of EECs, like Merkel cells, express the mechanosensitive channel Piezo2 to detect luminal forces and physical properties (Najjar & Margolis, 2022; Treichel et al., 2022). Previously, we systematically mapped the distribution of Merkel cells and sensory afferents in the murine and human oral cavity using modern histological methods (Moayedi et al., 2018, 2021). The hard palate and gums are densely populated with Merkel cell–neurite complexes, as well as Meissner's corpuscles, glomerular corpuscles, and free nerve endings. Merkel cells in both the palatine rugae and gingiva are innervated by sensory afferents, including neurofilament heavy + myelinated fibers, suggesting that these organs can mediate mechanosensory signal transduction. Indeed, cutaneous Merkel cells in whisker follicles and touch domes directly transduce mechanical stimuli (Hoffman et al., 2018; Ikeda et al., 2014; Maksimovic et al., 2014; Woo et al., 2014). Like cutaneous Merkel cells and EECs in GI tract, those in the oral epithelium express the mechanosensitive ion channel Piezo2 (Ikeda et al., 2014; Moayedi et al., 2018; Woo et al., 2014); however, the physiology of Merkel cells in oral epithelia has not been described. Whether these Merkel cells respond to mechanical stimulation is a fundamental open question. Given that Merkel cells encode discriminative touch in the skin, it is reasonable to postulate that Merkel cells in the oral mucosa are also mechanosensitive.

Here, we investigated the functional properties of Merkel cells in the oral cavity using a transgenic mouse line that expresses a calcium‐sensitive protein GCaMP6f (Chen et al., 2013) in Merkel cells. We developed a novel live‐cell imaging technique using ex vivo oral mucosa preparations to interrogate the responsiveness of Merkel cells to mechanical stimulation. With this recording system, we show that Merkel cells in gingiva had higher mechanical sensitivity than those in hard palate and also demonstrated that keratinocytes can also be activated by mechanical stimulation independent of Merkel cells. Through behavioral experiments, we demonstrated that mice lacking Merkel cells lose their preference for a mineral oil emulsion, suggesting the functional significance of Merkel cells in discriminating flavor quality. This study not only provides a novel technique capable of monitoring sensory responses in live oral mucosa tissues but also demonstrates that Merkel cells in epithelia displayed a range of physiological responses that may be crucial for detecting mechanical stimulation in the mouth.

## 2 METHODS

### 2.1 Animal use

All animal experiments were conducted with approval from and in accordance with policies of the Columbia University Institutional Animal Care and Use Committee and the NesTec internal review committee. Mice were maintained in a temperature‐controlled environment at Columbia University Medical Center with ad libitum access to food and water.

Table 1 summarizes genetic and protein biomarkers employed in this study. To label Merkel cells in oral cavity tissues, several transgenic mouse lines that drive the expression of genetically encoded reporters in Merkel cells were used. They included *J2XnGFP*, *VGLUT3* ^*Cre*^ *; Rosa26* ^*GCaMP6f*^, *VGLUT3* ^*Cre*^ *; Rosa26* ^*(GCaMP6f/tdTomato)*^, *VGLUT3* ^*Cre*^ *; Rosa26* ^*tdTomato*^, and *Atoh1* ^*CreERT2*^ *; Rosa26* ^*GCaMP6f*^. Among them, *VGLUT3* ^*Cre*^ *; Rosa26* ^*GCaMP6f*^ and *VGLUT3* ^*Cre*^ *; Rosa26* ^*(GCaMP6f/tdTomato)*^ mice express GCaMP6f in cells of the VGLUT3 lineage, including Merkel cells in gingiva and hard palate. *Atoh1* ^*CreERT2*^ *; Rosa26* ^*GCaMP6f*^ mice express GCaMP6f in *Atoh1*‐lineage cells following tamoxifen injection. These three mouse lines express GCaMP6f in Merkel cells whose internal calcium concentration can be easily monitored.

**TABLE 1:** Cell type‐specific markers and genetically encoded reporters.

Biomarker or transgene	Cell type marked or protein function	Note	References
*J2XnGFP*	*Atoh1* enhancer elements drive transgenic GFP expression in all Merkel cells	RRID: MGI:3836962	Lumpkin et al. (2003)
*Piezo2‐EGFP‐IRES‐Cre*	Piezo2–EGFP fusion protein expressed from endogenous *Piezo2* locus, marks Piezo2 protein localization	Jackson Laboratory; #027719, RRID: IMSR_JAX:027719	Woo et al. (2014)
*Atoh1* ^*CreERT2*^	Tamoxifen inducible Cre expressed from the endogenous Atoh1 locus. Used in this study to drive expression of reporter genes in newly specified and mature Merkel cells	RRID: MGI:3849175	Fujiyama et al. (2009)
*VGLUT3* ^*Cre*^	Cre expressed from the endogenous Vglut3 locus. Used in this study to drive expression of reporter genes in Vglut3 lineage cells, including mature Merkel cells	Also expressed by a subset of mechanosensory DRG neurons, including Merkel‐cell afferents Jackson Laboratory; #018147, RRID: MSR_JAX:018147	Grimes et al. (2011); Lou et al. (2013)
Keratin‐8 (K8)	Protein marker of mature Merkel cells that can be detected with antibody staining	Also expressed by taste cells	Toh et al. (1993); Vielkind et al. (1995)
GCaMP6f	Genetically encoded calcium‐sensitive fluorescent protein used for imaging cellular activity	Jackson Laboratory; #024105, RRID: IMSR_JAX:024105	Chen et al. (2013)
tdTomato	Genetically encoded red fluorescent protein used as a reporter for Vglut3 expression	Jackson Laboratory; #007908, RRID: IMSR_JAX:007908 Ai14 or #007909, RRID: IMSR_JAX:007909 Ai9	Madisen et al. (2010)

In addition to transgenic reporter mice, a limited set of imaging experiments used conditional knockouts (CKO) of the *Atoh1* gene (*K14* ^*Cre*^ *;Atoh1* ^*LacZ/Flox*^ mice, named *Atoh1* ^*CKO*^). *Atoh1* ^*CKO*^ mice completely lack Merkel cells (Morrison et al., 2009). *Piezo2* ^*EGFP‐IRES‐Cre*^ mice were used to identify cells expressing Piezo2 channels.

### 2.2 Ex vivo tissue preparation

**FIGURE 1:**
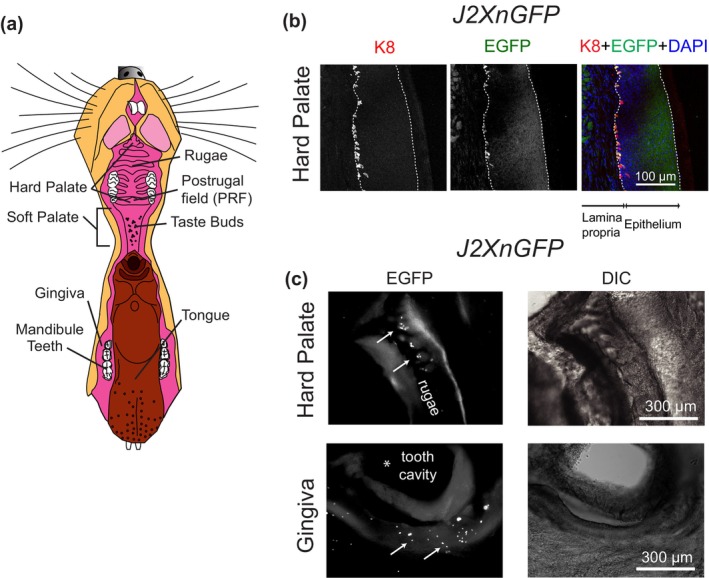
Identification of Merkel cells in oral mucosa. (a) The anatomy of the mouse oral cavity. (b) A representative cryosection of the hard palate from a J2XnGFP mouse, showed highly overlapped K8 and *Atoh1*‐EGFP staining. Dotted lines represent the boundaries between lamina propria, epithelium, and the edge of the tissue respectively. (c) Whole mount epithelial preparations showing EGFP+ Merkel cells and corresponding differential interference contrast (DIC) images. The upper panel shows representative images of a hard palate from a J2XnGFP mouse. The lower panel shows images of a gingival preparation. The marked (*) area in the GFP image corresponding with the bright area in the DIC photo is the region that surrounded a tooth (removed). Arrows indicate individual Merkel cells.

Figure 1a illustrates the anatomy of the oral cavity and indicates the relative location of hard palate and gingiva. Epithelial peel preparations were modified from our procedures for epidermis preparation (Hoffman et al., 2018). Palatal and gingival specimens including teeth and underlying bone were cut and dissected from the oral cavity of euthanized adult mice (1–4 months) and rinsed with extracellular recording solution which contained (in mM): 140 NaCl, 5 KCl, 10 HEPES (pH 7.4, adjusted with NaOH), 10 d‐glucose, 2 MgCl_2_, and 2 CaCl_2_. The palatal and gingival tissues were treated with dispase (Fisher Scientific; #354235)/HBSS (Thermo Fisher Scientific; #14175‐095) (1:1) on a shaker at room temperature for 1–1.5 h and 2–3 h, respectively. Palatal and gingival epithelia were then carefully detached from the lamina propria with forceps to expose the stratum basale. Calcium imaging experiments focused on the gingiva and the back of the hard palate, termed postrugal field (Kutuzov & Sicher, 1952; Nunzi et al., 2004), which contain high densities of Merkel cells and are relatively flat in ex vivo preparations (Moayedi et al., 2018). The anterior and middle parts of the hard palate were not tested because the undulating structure of rugae is not amenable to live‐imaging studies which require cells to be situated within the same focal plane following mechanical stimulation.

### 2.3 Calcium imaging

Physiological responses of oral epithelial cells following mechanical stimuli were measured using live‐cell imaging of two different calcium‐sensitive fluorescent indicators: GCaMP6f and Fura‐2. GCaMP6f, a genetically encoded, calcium‐sensitive GFP fusion with fast kinetics, was used to selectively monitor cytoplasmic calcium signaling in live cells (Chen et al., 2013). Fura‐2 is an exogenous ratiometric calcium indicator that is taken up across cell membranes through the form of Fura‐2 acetoxymethyl ester (Fura‐2 AM) (Grynkiewicz et al., 1985); therefore, the calcium responses of all epithelial cells including Merkel cells and keratinocytes could be imaged simultaneously. The Fura‐2 calcium imaging technique was modified by Hoffman et al. (2018).

After gingival/palatal epithelia expressing GCaMP6f+ cells were obtained, tissue was trimmed and placed inside a metal washer (inner diameter ~6 mm, thickness: ~1 mm) lightly glued on the bottom of the recording chamber. Tissues were positioned so that Merkel cells were against the bottom coverslip. The excess extracellular solution was gently removed with a Kimwipe. Low melting point agarose (~30 μL, 1.5%–2.0%, #16520‐100; Thermo Fisher Scientific) was first dissolved in extracellular solution, heated until melted, then cooled to 30–35°C before it was dropped onto the surface of the tissue to form an agarose block allowing for uniform mechanical stimulation of the specimen.

For tissues that lacked genetically encoded calcium indicators (*J2XnGFP* or *Atoh1* ^*CKO*^), Fura‐2 AM was used as the cytosolic calcium indicator. A mixture of Fura‐2 AM (Thermo Fisher Scientific; F‐1221, 20 μL, 250 μM) and Pluronic F‐127 (Thermo Fisher Scientific; P3000MP, 0.5%) was directly dropped onto the surface of the stratum basale of the epithelium. After 3 min, 1 mL extracellular solution was added, and tissues were kept in the solution for 30–45 min. The epithelial preparation was then carefully rinsed with extracellular solution 4–5 times to remove excess Fura‐2 AM and placed inside the metal washer. A Kimwipe was used to remove excess solution and low melting point agarose was used to embed the tissue. All procedures and experiments were performed at room temperature.

**FIGURE 2:**
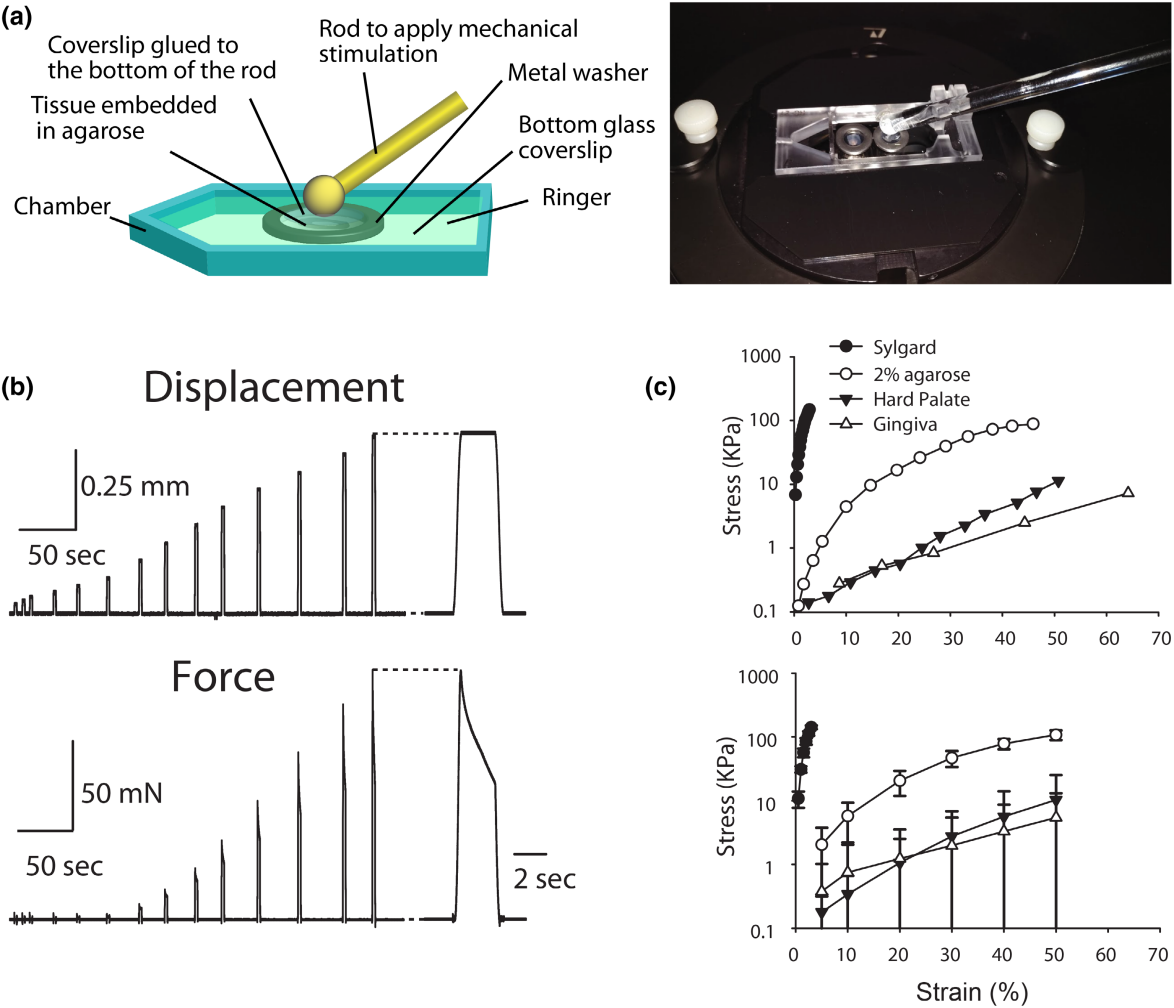
Experimental setup for calcium imaging on oral mucosa epithelia. (a) Left panel illustrates the setup to compress the embedded gingival or hard palatal epithelia and image activity. Right panel shows a photo of the setup. (b) Examples demonstrating the forces generated on the surface of an agarose block during a series of increased ramp‐and‐hold displacements to its surface. (c) Upper panel: Examples showing the stress–strain relationship of different materials and tissue preparations. Lower panel: Mean stress–strain relationship from nine experiments. Error bars represent SD. *N* = 5 hard palate, 9 gingiva, 4 agarose, and 2 Sylgard experiments.

GCaMP6f and ratiometric calcium imaging were performed using an Olympus inverted microscope IX81 equipped with an Olympus UApo 20×/0.75 objective. Controlled mechanical stimuli were applied to the top surface of the agarose block. Chroma 49002 filter set (ET470/40X, T495lpxr and ET525/50m) was used for single‐wavelength, blue light GCaMP imaging. Mechanical stimuli were applied by a small piece of round coverslip glued to the bottom of a rod (Figure 2a) controlled by a Sutter micromanipulator. The stimuli were applied briefly (~1 s) and released to allow the compressed tissue to return to its original focal plane. Time‐lapse images were collected using MetaFluor 7.5.6.0 and analyzed in Fiji ImageJ 2.0. A Chroma 49008 filter set (ET560/40X, T585lpxr and ET630/75m) was used for tdTomato fluorescence imaging. For Fura‐2‐loaded cells, fluorescence was excited by two wavelengths 340 and 380 nm, respectively, as the ratio of the fluorescence emission at these two wavelengths correlates with the cytosolic‐free calcium concentration. Frame rates were 0.8 s for GCaMP6f and 1.2 s for Fura‐2. To compensate for hysteresis, individual images were aligned using the ImageJ Template Matching plugin (https://sites.google.com/site/qingzongtseng/template‐matching‐ij‐plugin) before analysis (Tseng et al., 2011, 2012). In GCaMP6f experiments, regions of interest (ROIs) were drawn to encircle single‐cell bodies (diameter: ~9–12 μm). In Fura‐2 experiments, ROIs were drawn to cover only the center region of the cells (diameter ~3–4 μm) to reduce signal contamination from immediate neighboring cells. This was less of a problem in GCaMP6f experiments due to low baseline fluorescence and high GCaMP6f+ cell spacing. Mean gray values of individual ROI were measured using ImageJ. To compensate for photobleaching of the fluorescence in GCaMP6f experiments over time, the intensity of the background signal from the no‐cell area of comparable ROI size was subtracted from the calcium signal intensities, the resulting signal intensities (*F*_t_) was then normalized to the signal intensity at *t*_0_ (*F*_0_): Δ*F*/*F* = (*F*_*t*_ − *F*_0_)/*F*_0_. Images that were out of focus during or immediately after displacement were not analyzed (1–3 frames). To compare the calcium responses between different tissues, several parameters were measured and calculated using custom Matlab code. They are defined as follows: time‐to‐peak: the time from the initial stimulus artifact to the peak of the recorded response; rising slope: the highest slope of the rising phase; *τ*_decay_: time from the peak to 50% of the peak, *τ*_decay_ >70 s were set as 70 s comparing the *τ*_decay_ from different tissues; delay of the response: time from the stimulus artifact to 10% of the peak intensity.

For experiments using a high KCl solution to activate Merkel cells in epithelial tissues, an Olympus upright microscope BX61WI equipped with an Olympus XLUMPlanFLN 20×/0.95 W objective was used. Epithelial tissues were glued with Loctite® super glue on the bottom of a slice recording chamber with Merkel cells facing up. GCaMP6f calcium‐imaging experiments were done using the Chroma 49002 filter set (ET470/40X, T495lpxr, ET525/50m). tdTomato fluorescence was recorded using 49008 filter set (ET560/40X, T585lpxr and ET630/75m).

### 2.4 Mechanical property measurement of agarose and tissue preparations

To measure the mechanical properties of epithelia, agarose, and Sylgard respectively, strain and stress relationships were first established. Ramp‐and‐hold displacements (0.1–1.3 mm) were delivered with a custom‐built indenter probe (1.55 mm ceramic tip; 3–5 s hold phases). The applied displacements were commanded using a model XPS motion controller and driver system (Newport) connected to a PC computer (Maksimovic et al., 2014). The movement of the indenter was controlled with custom‐made software and measured with a laser distance‐measuring device (OptipNCDT 1402; Micro‐Epsilon). The period between successive displacements was 30 and 60 s. The forces applied on the surface of the material were measured, digitized, and recorded using Sci‐Works Experimenter software (DataWave Technologies). Epithelial preparations or 2% agarose blocks fit into metal washers was placed on the top of a Sylgard (Sylgard 184; Dow Corning, Inc., curing at room temperature) coated petri dish. Sylgard is >100 times more rigid than 2% agarose according to the stress–strain relations (see Section 3), thus the Sylgard dish bottom would not interfere with the measurement of the mechanical properties for agarose and tissue preparations.

To establish the relationship between the displacement and the force, the position of the indenter was adjusted close to the surface of the material and then a series of ramp‐and‐hold displacements were delivered. Following data collection, the “ideal” surface position of each measured agarose or tissue was calculated by extrapolating two force measurements closest to zero and then defined as zero displacement for later displacement calculation. The strain and the stress of the compressed agarose/tissues are defined follows:

$$\begin{array}{c} \text{Strain}\;\left(\%\right)=\text{displacement}\;(\text{against the}\;“\text{ideal}\;{\text{surface}}^{”})\\ \;/\text{thickness of sample}\;\left(\text{agarose or epithelium}\right), \end{array}$$

$$\begin{array}{c} \text{Stress}\;\left(\mathrm{kPa}\right)=\text{force}\;\left(\text{newton}\right)\\ \;/\text{surface area of the indenter}\;\left(m^{2}\right). \end{array}$$

### 2.5 Tamoxifen administration

For experiments involving *Atoh1* ^*CreERT2*^ *;Rosa26* ^*GCaMP6f*^ mice, 1 dose (100 mg/kg) of tamoxifen was administered by intraperitoneal injection. GCaMP6f expression was observed 1–2 weeks following tamoxifen injection (see Section 3).

### 2.6 Immunohistochemistry

For whole‐mount epithelial tissue staining, palatal and gingival specimens were fixed in 4% paraformaldehyde at 4°C overnight. After fixation, tissues were washed in phosphate buffer saline (PBS) and then incubated in 5% NGST blocking solution (5% normal goat serum, 0.1 M PBS, 0.3% Triton X‐100) for 1 h, and then in 1% NGST buffer (1% goat serum, 0.1 M PBS, 0.3% Triton X‐100) containing primary antibodies for 12–36 h. Tissue was then washed 3× in 1% NGST and transferred to 1% NGST containing secondary antibody for 2 h. Tissues were then washed with 0.1 M phosphate buffer three times and embedded in Fluoromount‐G with DAPI (Southern Biotech). Rat anti‐Keratin 8 (K8; DSHB, 1:100, TROMA1‐s, RRID: AB_531826), goat anti‐GFP‐FITC (Abcam; Ab6662, 1:500, RRID: AB_305635), chicken anti‐GFP (Abcam; Ab13970, 1:500, RRID: AB_300798), anti‐K14 (Biolegend; 906004, 1:250, RRID: AB_2616962) were used as primary antibodies. Secondary antibodies included Thermo Fisher (Invitrogen) goat anti‐chicken Alexa Fluor 488 (#A‐11039, RRID: AB_2534096), Alexa Fluor 647 (#A‐21449, RRID: AB_2535866), goat anti‐mouse Alexa Fluor 594 (#11032, RRID: AB_2534091), goat anti‐rabbit Alexa Fluor 647 (#21244, RRID: AB_2535812), Alexa Fluor 594 (#A11012, RRID: AB_2534079), anti‐rat Alexa Fluor 647 (#A‐21247, RRID: AB_141778) and Alexa Fluor 594 (#A‐11007, RRID: AB_10561522).

For cryosection tissue staining, palatal tissue was fixed for 2 h in 4% PFA at room temperature, washed in PBS, and then decalcified for 1–2 weeks in 10% ethylenediaminetetraacetic acid pH 7.4 at 4°C on a rotary mixer. When soft, tissue was cryoprotected in 30% sucrose overnight at 4°C and then embedded in Tissue‐Tek OCT over liquid nitrogen. Cryosections of 25 μm thickness were mounted onto Superfrost slides (Fisherbrand). Slides were then kept at 37°C for 45 min to 3 h and prehybridized in PBS containing 5% normal goat serum and 0.3% Triton X‐100. Slides were then hybridized overnight at 4°C with primary antibodies diluted in a hybridization solution. After three washes in PBS with 0.3% Triton X‐100, slides were incubated with secondary antibodies diluted in a hybridization buffer for 45 min to 2 h, washed three times in PBS, and embedded in Fluoromount‐G with DAPI (Southern Biotech). Chicken anti‐GFP (Abcam; ab13970, 1:1000, RRID: AB_300798) and rat anti‐Keratin 8 (K8; DSHB, 1:100, TROMA1‐s, RRID: AB_531826) were used as primary antibodies.

Images were acquired on a laser scanning confocal microscope (Carl Zeiss LSR Exciter) with Plan‐Neofluar 10×/0.3, Plan‐Apochromat 20×/0.8, or EC Plan‐Neofluar 40×/1.3 objectives or an Olympus epifluorescence inverted microscope IX81 equipped with UApo 20×/0.75 objective. ImageJ was used to estimate the covering area of GCaMP6f+ cells in a specific region of epithelial tissues from *Atoh1* ^*CreERT2*^ *;Rosa26* ^*GCaMP6f*^ mice.

### 2.7 Behavior

Transgenic *Atoh1* ^*CKO*^ mice along with littermate controls were tested for the ability to discriminate a mineral oil emulsion (concentration range: 0%–20% mineral oil emulsified with 0.75% Tween‐80, droplet size <5 μm diameter). Singly housed mice were weighed and then placed on water restrictions for 22.5 h. On each testing day, mice were water restricted for up to 22.5 h, tested for 30 min, and then allowed 1 h of free access to water. This water restriction protocol has been shown in the literature to be tolerable in mice without significant weight loss (Glendinning et al., 2002). Mice maintain 85%–90% of baseline weight. During testing, single mice were placed in a cage equipped with a Davis rig gustometer to record licking behavior (Smith et al., 1992). This apparatus has a brief access window that is shuttered to control the timing of liquid availability. On the first training day, mice were placed in the testing chamber with the window fully open for 30 min to allow familiarization with the sipper spout. On the second training day, the shutter would close 5 s after the first lick and would remain closed for 7.5 s before re‐opening. On this day, all trials used normal water, and the total session was 30 min. This training was repeated on day 3 to ensure that mice were familiar with the apparatus and licked readily. If mice failed to learn how to use the spout on day 3, they were given additional training days until they performed the task. After training day 3, mice were given unrestricted food and water access for 1 h, then placed on a food and water restriction overnight (1 g food, 2 mL water). On the fourth day, during the 30‐min testing sessions, the shutter was programmed to allow mice 5‐s access to liquid after the first lick. The shutter then closed, cutting off access while the water bottle was changed. After a 7.5‐s wait period, the shutter reopened for the next trial. During testing, six different concentrations of solution were tested (0%–20% mineral oil emulsion). If mice failed to complete at least 12 trials during the testing session, they were removed from analysis. 4/23 Control and 7/20 *Atoh1* ^*CKO*^ mice did not complete 12 trials. The cumulative number of licks for mineral oil emulsion of each concentration during each 30‐min trial was counted as an indicator of their mineral oil preference. After each testing day, mice were weighed to determine whether they maintained >80% of their baseline weight.

### 2.8 Statistics

All data are given as mean ± SD. Differences between groups were assessed by unpaired two‐tailed Student's *t* test when both groups of samples followed the KS normality test. For data that failed normality testing, a two‐tailed Mann–Whitney test was used. Statistical analyses were performed using GraphPad Prism 5.0.

## 3 RESULTS

### 3.1 Identification of Merkel cells in ex vivo oral mucosa preparations

To investigate the physiological responses of oral Merkel cells, we first established semi‐intact oral epithelial preparations for live‐cell imaging. To visualize Merkel cells in situ, we used transgenic mice in which nuclear‐localized EGFP is driven by transcription factor Atoh1 enhancer sequences (*J2XnGFP*) (Lumpkin et al., 2003). EGFP+ cells were located between the lamina propria and epithelium in the hard palate (Figure 1a,b). To confirm that EGFP+ cells were Merkel cells, the hard palate was stained with anti‐keratin 8 (K8), a Merkel‐cell marker, and anti‐GFP antibodies. The majority of EGFP+ cells in the hard palate expressed K8+, demonstrating that these EGFP+ cells in hard palate are Merkel cells.

In oral epithelia, K8+ Merkel cells are localized at the basal layer of the epithelium adjacent to the lamina propria. This localization poses a problem for live‐cell imaging as the thickness of both the epithelium and lamina propria cause optical interference. To overcome this problem, epithelium was separated from lamina propria to expose the Merkel cells using dispase digestion (see Section 2). The epithelium was then embedded in agarose with Merkel cells facing the coverslip. Individual Atoh1‐EGFP+ cells were clearly distinguishable in hard palate and gingiva using an inverted microscope (Figure 1c), confirming that Merkel cells were preserved in epithelial tissues following separation from the lamina propria. To establish an ex vivo Merkel cell stimulation system capable of mimicking the pressure sensed by the oral cavity epithelia, we adapted a commercial chamber (RC‐27; Warner Instruments, Section 2) as shown in Figure 2a.

Given that ex vivo tissue preparations were positioned in agarose, the elasticity of the agarose might lead to misestimation of applied mechanical stimulation on tissues if they have a comparable or lower elasticity than agarose. We thus estimated the elasticity of the tissue to ascertain whether agarose could skew the pressure stimuli applied to mucosal preparations. We used a custom indenter to establish the relationship between stress and strain by measuring the displacement of the agarose/epithelial preparations and the generated compressive force. An increase in displacement was accompanied by an increased force once the indenter touched the surface of the agarose (Figure 2b). The derived strain–stress relationship of oral tissues and agarose block was not linear (Figure 2c); therefore, Young's modulus could not be calculated in a straightforward manner. We thus used the stress required to reach a percentage of strain (5%–50% in our study, corresponding to the displacement of ~60–600 μm for 1.2 mm agarose thickness) for Merkel cell activation to compare the relative elasticity of different materials. Both agarose and epithelial tissues were placed on a Sylgard‐coated dish for these tests. Based on our data, the stress required to reach the same strain in 2% agarose was an order of magnitude higher than that of gingival or palatal epithelia (two‐way ANOVA, *p* < 0.001) demonstrating that agarose is compliant compared with tissue elasticity. Meanwhile, the elasticity of Sylgard was >100 times higher than that of 2% agarose, demonstrating that our measurements would not be affected by the elasticity of Sylgard. These data also show that the elasticity of hard palate and gingival epithelia were not significantly different (two‐way ANOVA, *p* = 0.35) and most of the applied compression transmitted to epithelial tissues was not dominated by agarose.

### 3.2 Merkel cells from ex vivo preparations responded to mechanical stimulation

We next tested whether mechanical stimulation activates Merkel cells in ex vivo epithelial preparations using the imaging preparations just described.

Calcium imaging can simultaneously monitor the activity of populations of cells. The genetically encoded calcium sensor family of GCaMP proteins has been used extensively to monitor neuronal activity in different tissue preparations (Broussard et al., 2014). We took advantage of the Cre/*loxP* system to express GCaMP6f in Merkel cells using two mouse lines, *VGLUT3* ^*Cre*^ (Grimes et al., 2011) and *Atoh1* ^*CreERT2*^ (Machold & Fishell, 2005) as the vesicular glutamate transporter VGLUT3 and the transcription factor Atoh1 are both expressed by Merkel cells (Lou et al., 2013; Lumpkin et al., 2003; Nunzi et al., 2004). To validate whether GCaMP6f was selectively expressed in epithelial Merkel cells in *VGLUT3* ^*Cre*^ *;Rosa26* ^*GCaMP6f*^ *and Atoh1* ^*CreERT2*^ *;Rosa26* ^*GCaMP6f*^ mice, we performed immunohistochemical staining on gingival and hard palatal epithelia.

**FIGURE 3:**
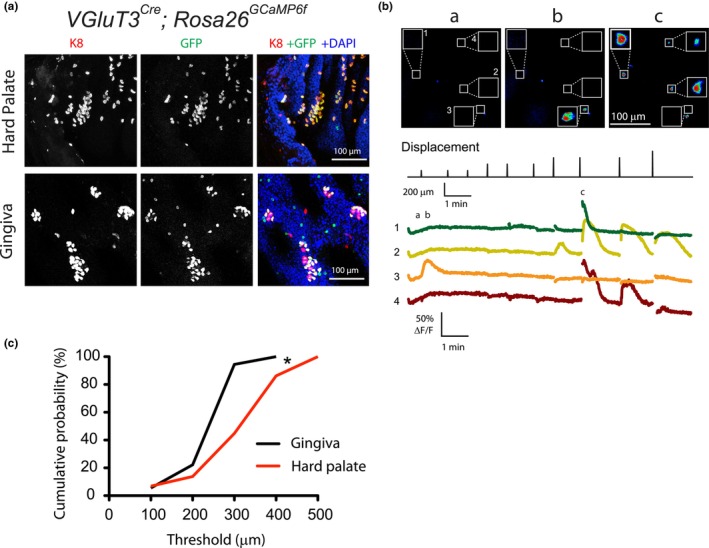
Merkel cells in oral mucosa were activated by mechanical stimuli while gingiva exhibited lower threshold for their activation compared with those in hard palate. (A) Representing images showing colocalization of GCaMP6f and the Merkel‐cell marker K8 from a *VGLUT3* ^*Cre*^ *;Rosa26* ^*GCaMP6f*^ mouse. K8 and calcium indicator GCaMP6f were highly colocalized in the hard palate. In gingiva, only clustered GCaMP6f+ cells colocalized with K8. Most of the scattered GCaMP6f+ cells did not show K8 staining. (B) An example of cytosolic calcium increases in GCaMP6f+ Merkel cells following brief mechanical stimuli. The upper panel shows fluorescence images of GCaMP6f+ cells at different time frames (a–c), which correspond to the time points (a–c) in the lower panel showing calcium responses in Merkel cells from a *VGLUT3* ^*Cre*^ *;Rosa26* ^*GCaMP6f*^ mouse. Four insets (1–4) in the upper panel are enlarged images of cells 1–4 exhibiting increased fluorescence. The middle panel shows individual displacements. (C) A cumulative probability figure illustrating the summary of Merkel cell activation thresholds from gingiva and hard palate. *A significant difference between two populations of the threshold (*p* < 0.005 for the two‐tailed Mann–Whitney test).

Gingival and hard palatal epithelia from *VGLUT3* ^*Cre*^ *;Rosa26* ^*GCaMP6f*^ mice were stained with anti‐GFP and anti‐keratin 8 (K8) antibodies to label GCaMP6f+ and mature Merkel cells, respectively. As expected, most of the GCaMP6f+ cells in the hard palate showed robust expression of the Merkel‐cell marker K8 (97%, *n* = 182 cells from 4 animals). Likewise, the majority of K8+ cells also showed GCaMP6f expression (91%, *n* = 193 cells from four animals) (Figure 3A). These data demonstrate that most hard palatal GCaMP6f+ cells are Merkel cells and most cells that do not express GCaMP6f protein are not Merkel cells. In gingival epithelium, GCaMP6f+ cells could be classified into clustered and scattered cells according to their spatial distribution. Most of the GCaMP6f+ cells found in clusters also expressed K8 (96%, *n* = 128 cells from three animals) and 98% of K8+ cells expressed GCaMP6f (total *n* = 126 cells from three animals). Surprisingly, only 22% of scattered GCaMP6f+ cells expressed K8 (*n* = 149 cells from five animals), suggesting that the majority of the scattered GCaMP6f+ cells were not typical, mature Merkel cells. It is possible that these scattered GCaMP6f+ cells are newly differentiated Merkel cells that have not yet turned on K8 expression, as the onset of K8 expression always follows *Atoh1* expression (Morrison et al., 2009). Alternatively, they might represent a subpopulation of non‐Merkel cells that are *VGLUT3*‐lineage. Together, these data validated *VGLUT3* ^*Cre*^ *;Rosa26* ^*GCaMP6f*^ mice as a reliable mouse line for imaging Merkel cells in hard palate and gingiva.

We also examined the oral cavity tissues from a second mouse line, *Atoh1* ^*CreERT2*^ *;Rosa26* ^*GCaMP6f*^ as this *Atoh1* ^*CreERT2*^ transgenic line has been reported to drive selective expression of reporters in epidermal Merkel cells (Feng et al., 2018; Wright et al., 2015). We performed a tamoxifen injection on two mice (100 mg/kg per injection) and waited for 6 and 13 days (Figure S1A), respectively, to examine whether *Atoh1* ^*CreERT2*^‐lineage cells expressed the Merkel‐cell marker K8. GFP immunostaining from both mice revealed reporter expression widespread in oral epithelial cells, displaying cells with different morphologies, sizes of regions, and various degrees of GFP intensity. With K8 immunohistochemical staining, only 30 ± 16% of K8+ Merkel cells displayed GFP expression (128/433 K8+GFP+/K8+ cells, 11 regions, 4 epithelial tissues, *n* = 2 mice, Figure S1B). It was difficult to count the number of GFP+ cells in a specific region as the boundary of these cells could not be clearly defined (Figure S1B(e,h)). We, therefore, estimated the expression of GCaMP6f+ from *Atoh1* ^*CreERT2*^ *;Rosa26* ^*GCaMP6f*^ mice based on the fluorescent area coverage. GCaMP6f expression covered 0.2%–14% of the epithelial tissues (5.1% ± 4.2%, 11 regions, 4 epithelial tissues from 2 animals) of the epithelia, in which 0%–21.7% (8.5% ± 7.2%) of the GFP+ region displayed K8 fluorescence. These data suggest that many *Atoh1* ^*CreERT2*^‐lineage cells were not Merkel cells (Figure S1C). These GCaMP6f+/K8‐ cells were likely located within the epithelial layer (data not shown), including prickle and basale layers, indicating that they may be keratinocytes derived from an Atoh1 lineage, Merkel‐cell progenitors or immature Merkel cells (Woo et al., 2010). These Atoh1‐lineage K8‐negative cells were also observed in epidermis (data not shown), demonstrating they are not limited to oral cavity tissues. The cells from this mouse line were therefore excluded from further analysis because their identities as mature Merkel cells could not be confirmed. Thus, we conclude that *VGLUT3* ^*Cre*^ *;Rosa26* ^*GCaMP6f*^ mouse line is a better choice than the *Atoh1* ^*CreERT2*^ *;Rosa26* ^*GCaMP6f*^ for the study of Merkel‐cell physiology in oral mucosa.

We next prepared ex vivo gingival and palatal epithelium preparations from *VGLUT3* ^*Cre*^ *;Rosa26* ^*GCaMP6f*^ mice for live‐cell imaging of Merkel‐cell activity in response to mechanical stimulation. Cytoplasmic calcium signaling was monitored as a proxy for cellular excitability because Merkel cells have robust voltage‐activated calcium channels and internal calcium stores that convert cellular excitation into fast calcium increases (Haeberle et al., 2008; Hoffman et al., 2018; Ikeda et al., 2014; Piskorowski et al., 2008). Healthy Merkel cells had low resting calcium levels, and thus low GCaMP6f fluorescence intensity in the absence of touch. Test compressions were applied to the tissue to locate mechanically sensitive Merkel cells. After identifying a few responding cells, calcium activity was imaged in response to calibrated and predefined displacements. Data were collected from 18 epithelial preparations from 9 *VGLUT3* ^*Cre*^ *;Rosa26* ^*GCaMP6f*^ mice. GCaMP6f+ cells in both gingiva and hard palate showed a range of responses to mechanical stimuli, including different response kinetics and mechanical thresholds (Figure 3B). Most cells displayed immediate responses following mechanical stimuli, whereas some cells showed delayed responses (Figure S2a). A handful of GCaMP6f+ cells showed prolonged, nonrecovery responses that are typical of damaged cells and were not included in the analysis. With each interstimulus interval of 45–90 s, ~47% (34/73) of the responding GCaMP6f+ cells could be repeatedly activated and ~53% (39/73) of them were activated only once.

Compression resulted in Merkel cells being displaced out of focus for 1–3 frames following stimulation. Given that the GCaMP6f imaging frame rate was ~0.8 s/frame, some calcium responses showing fast rising time were not fully captured (e.g., Figure 3B, cell 1, time c). Despite this limitation, the rising and decay phase of 70%–75% Merkel cells could be fully captured under these experimental conditions. All of the calcium responses with slower rising time were fully captured (e.g., Figure 3B, traces 2, 3). These data suggest that there might be distinct physiological responses with different rise times in different Merkel cells. To explore this, the time‐to‐peak of the calcium responses immediately following agarose compression was analyzed and compared. As shown in Figure S2b, no clear separation between fast and slow responses was found in both gingiva and hard palate, suggesting that time‐to‐peak may not be a good criterion to categorize Merkel cell responses. Time‐to‐peak of the calcium responses in gingiva and hard palate were also compared, but no significant difference was found (median 6.4 s, *n* = 20 vs. 8.7 s, *n* = 41; two‐tailed Mann–Whitney test, *p* = 0.59). Because some of the cells showed delayed responses following displacements (median delay time: 1.4 s, *n* = 20 vs. 1.4 s, *n* = 41; two‐tailed Mann–Whitney test, *p* = 0.15), the time‐to‐peak would not correctly represent the rising rate of the responses. We, therefore, analyzed the slope of the rise phase, with the unit of the fluorescence percentage change (Δ*F*/*F*, %) divided by seconds (%/s). The mean rising slopes from gingival and palatal Merkel cells were not significantly different (median: 22%/s, *n* = 20, vs. 19%/s, *n* = 41, *p* = 0.54; two‐tailed Mann–Whitney test, Figure S2c). Decay kinetics of mechanically evoked responses were also widely distributed, as shown in Figure S2d, and may reflect the activity differences of voltage‐activated calcium and potassium channels (Piskorowski et al., 2008). No significant difference in decay constants was discovered between gingival and hard palatal Merkel cells (6.4 s, *n* = 20 vs. 8.7 s, *n* = 41, *p* = 0.29; two‐tailed Mann–Whitney test). Gingival and hard palatal Merkel cells also showed similar peak calcium response intensity (53%, *n* = 20 vs. 75%, *n* = 41, *p* = 0.10, two‐tailed Mann–Whitney test).

Interestingly, with increased displacement steps applied to the oral mucosal epithelia, Merkel cells in gingiva displayed a significantly lower mean threshold, and thus, higher mechanical sensitivity, than those in hard palate (median threshold: 300 μm, *n* = 18 vs. 400 μm, *n* = 29, *p* < 0.005, two‐tailed Mann–Whitney test, Figure 3C). Scattered GCaMP6f+ *VGLUT3*‐lineage nontypical Merkel cells in gingiva also showed a similar median threshold as gingival Merkel cells (300 μm, *n* = 6, *p* = 0.25, two‐tailed Mann–Whitney test). Quantitatively, the thresholds to activate most Merkel cells in oral mucosa ranged from 100 to 500 μm, comparable to the range (0.4–1.6 mm) which activated tactile afferents in intact skin preparations (Maksimovic et al., 2014), and corresponding to ~8%–40% agarose strain and ~5–75 kPa compressive pressure (Figure 2c).

**FIGURE 4:**
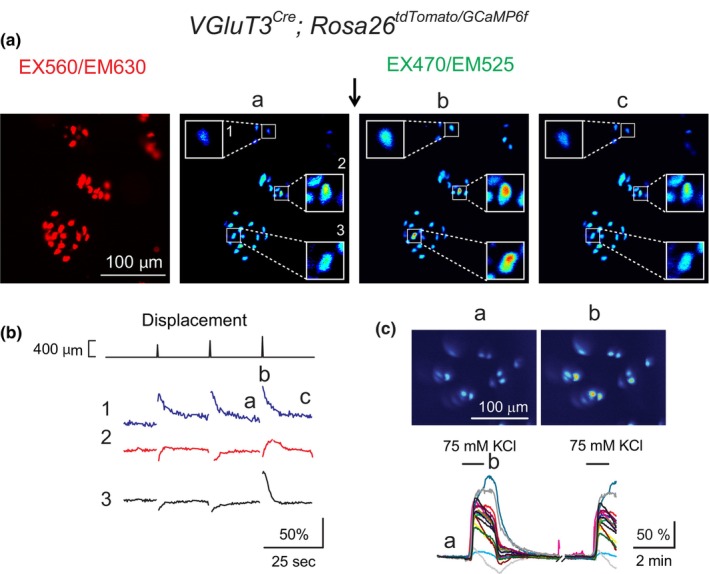
A substantial number of Merkel cells from oral epithelial preparations were not activated by mechanical stimulation. (A) Left panel displays representative tdTomato+/GCaMP6f+ Merkel cells in gingival preparation from a *VGLUT3* ^*Cre*^ *;Rosa26* ^*(tdTomato/CaMP6f)*^ mouse using tdTomato fluorescence filter set EX560/EM630. Panels (a–c) are the time series of the fluorescence changes of the same cells under EX470/EM525, a typical GFP fluorescence filter set. The arrow indicates a tissue compression. Three squares indicate cells 1–3 exhibiting increased fluorescence. Due to bleed‐through of the tdTomato fluorescence, Merkel cells in these preparations showed some fluorescence baseline in Merkel cells under EX470/EM525 filter set. (B) Time course of the displacement‐induced calcium responses. Numbers 1–3 and time (a–c) correspond to cells 1–3 and calcium responses time points (a–c) in (A). (C) A representative example shows high KCl superfusion reproducibly activated virtually all Merkel cells in hard palatal epithelium. Upper panels represent GCaMP6f fluorescence images before and during high KCl application. Lower panel is the time course of KCl‐induced calcium responses in hard palatal Merkel cells.

It is of interest that a substantial number of Merkel cells were not activated in our experimental protocol. To estimate the percentage of Merkel cells activated under these conditions, we generated *VGLUT3* ^*Cre*^ *;Rosa26* ^*GCaMP6f/tdTomato*^ mice which express both GCaMP6f and tdTomato in *VGLUT3*‐lineage cells. All resting GCaMP6+ cells were easily visualized by imaging tdTomato fluorescence. Surprisingly, unlike earlier experiments where GCaMP6f+ Merkel cells from *VGLUT3* ^*Cre*^ *;Rosa26* ^*GCaMP6f*^ mice showed virtually no visible baseline fluorescence without activation (Figure 3B), all tdTomato+ cells from *VGLUT3* ^*Cre*^ *;Rosa26* ^*GCaMP6f/tdTomato*^ mice displayed clear baseline fluorescence in the green fluorescence channel (Figure 4A). This enhanced baseline fluorescence was likely due to bleed‐through of red fluorescence as a similar fluorescence baseline was also observed in Merkel cells from *VGLUT3* ^*Cre*^ *;Rosa26* ^*tdTomato*^ oral cavity epithelia under green fluorescence channel (data not shown). To test if these Merkel cells in oral cavity were unhealthy cells due to high tdTomato expression, we directly superfused high KCl solution (75 mM) to the epithelial tissues from *VGLUT3* ^*Cre*^ *;Rosa26* ^*GCaMP6f/tdTomato*^ mice and measured calcium responses from Merkel cells. Virtually every Merkel cell showed robust calcium response following high KCl application (three epithelia, Figure 4C), suggesting that the majority of GCaMP6f+ Merkel cells were healthy and responding cells.

In spite of the higher fluorescence background, mechanical stimulation still increased the fluorescence intensity in some Merkel cells from *VGLUT3* ^*Cre*^ *;Rosa26* ^*GCaMP6f/tdTomato*^ epithelia (Figure 4A,B), demonstrating that these Merkel cells responded to mechanical stimulation. From a total 31 tdTomato+ Merkel cells imaged from two mice, six cells showed increased GCaMP6f fluorescence following tissue compression, suggesting that ~20% of Merkel cells were activated with this stimulation paradigm. It is possible that the stimulus range was not sufficient to elicit detectable signals under these imaging conditions, or no efficient transmission of the major pressure from the glass coverslip to individual Merkel cells in epithelia. Alternatively, some Merkel cells might have been immature and thus unresponsive to mechanical stimuli. It is also possible that mechano‐insensitive Merkel cells lack PIEZO2 ion channels (Moayedi et al., 2018).

**FIGURE 5:**
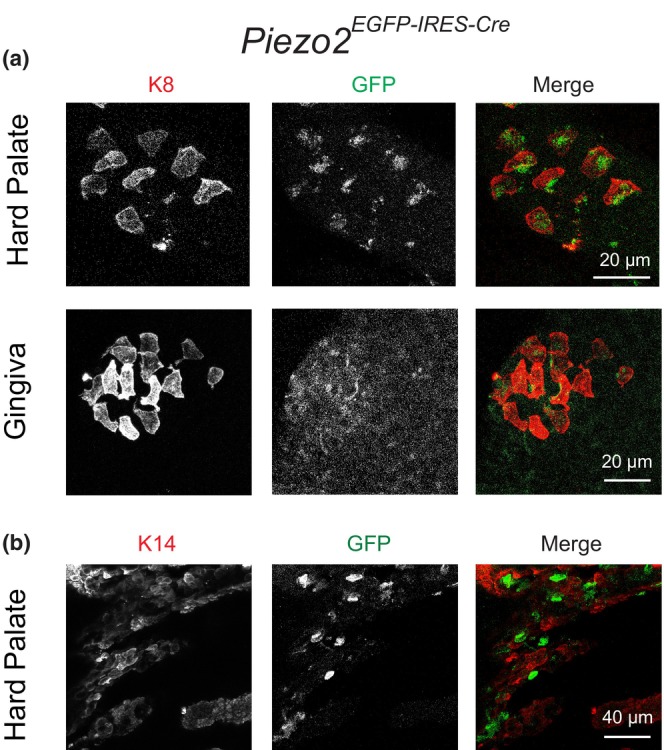
Piezo2 channels were expressed at or near the side of Merkel cells in hard palate and gingiva. (a) Examples show K8 and GFP immunoreactivity of hard palatal and gingival epithelium from a *Piezo2* ^*EGFP‐IRES‐Cre*^ mouse. Note that the Piezo2 channels are preferentially located on one side of the cells. (b) An example shows costaining of keratinocyte marker K14 and GFP of gingival epithelium. Note that most of the GFP+ Piezo2‐expressing cells were not labeled by K14.

To test this possibility, we performed whole‐mount immunohistochemical staining of gingival and hard palatal epithelia from *Piezo2* ^*EGFP‐IRES‐Cre*^ mice. In these experiments, 95% and 84% of K8+ Merkel cells from gingiva and hard palate, respectively, showed Piezo2‐EGFP immunoreactivity (39/41 K8+ cells in gingiva, 69/82 K8+ cells in hard palate, from two *Piezo2* ^*EGFP‐IRES‐Cre*^ mice; Figure 5a), indicating that a large majority of Merkel cells have PIEZO2 ion channels. In some cases, Piezo2+ immunoreactivity was juxtaposed to K8+ immunoreactivity, suggesting that some Piezo2 channels are localized at nerve terminals innervating Merkel cells. Interestingly, some K8‐negative epithelial cells also highly expressed Piezo2, consistent with our earlier report (Moayedi et al., 2018). Costaining of the epithelia with keratinocyte marker K14 and GFP antibodies indicated separate populations of K14+ and Piezo2+ cells (Figure 5b), suggesting that some PEIZO2+ mechanosensitive cells are not keratinocytes.

### 3.3 Activation of Merkel cells and non‐Merkel cells in oral mucosa

**FIGURE 6:**
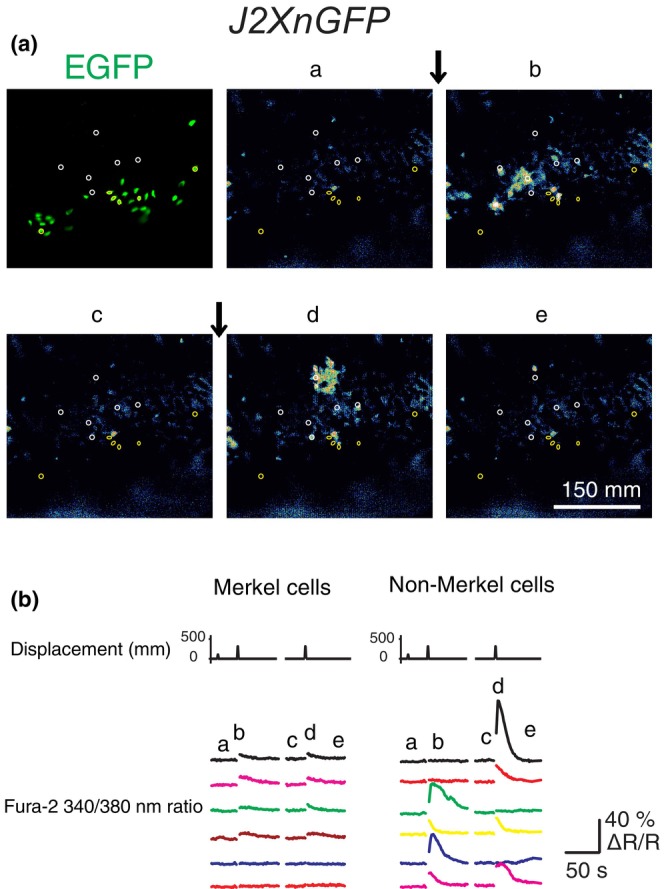
Both Merkel and non‐Merkel cells in oral mucosa were activated by mechanical stimulation. (A) Upper left photo illustrates EGFP+ Merkel cells in a gingival epithelium from a J2XnGFP mouse under EX470/EM525 filter. (a–e) Fura‐2 ratiometric images of the same preparation at different time points. Yellow and white circles indicate Merkel and non‐Merkel cells respectively. The vertical arrows indicate the time of tissue compressions. (B) Calcium responses of individual Merkel cells and non‐Merkel cells following physical displacements. (a–e) correspond to different time points in (A). The fluorescence ratio changes (∆*R*) are further normalized to their baseline fluorescence ratios (*R*).

Although some Merkel cells in gingiva and hard palate were activated following mechanical stimulation, it is not clear if Merkel cells are the only cell types responsive to mechanical stimulation. To answer this question, calcium responses following displacement from the entire epithelium were recorded using Fura‐2 in mice expressing nuclear GFP in Merkel cells (J2XnGFP). Hard palatal and gingival epithelia obtained from J2XnGFP mice were first isolated, loaded with Fura‐2, and then embedded in agarose (see Section 2). Calcium responses from epithelial cells expressing EGFP and those that lack EGFP in the gingival and hard palatal preparations could be identified independently by overlaying EGFP+ fluorescence with Fura‐2 ratio images (Figure 6A). In 125 EGFP+ cells analyzed, 25 (20%) of them responded to mechanical stimulation, comparable to the percentage found in GCaMP6f experiments from *VGLUT3* ^*Cre*^ *;Rosa26* ^*GCaMP6f/tdTomato*^ epithelia.

Surprisingly, mechanical stimulation also triggered increases in Fura‐2 fluorescence in many epithelial cells that were not Merkel cells. Robust calcium signals propagated throughout the epithelia like calcium waves (Tsutsumi et al., 2009). These unexpected results suggest that non‐Merkel epithelial cells also have the potential to encode mechanical stimuli. More surprisingly, these epithelial cells showed three to four times stronger internal calcium increases compared to Merkel cells following mechanical stimuli as shown in Figure 6B (∆340/380 Ratio, mean ± SD: 0.36 ± 0.19, *n* = 25 vs. 0.12 ± 0.07, *n* = 25 for non‐Merkel and Merkel cells, respectively, *p* < 0.0001, two‐tailed *t* test). These weaker calcium responses in Merkel cells might be due to confounding ratiometric imaging of Fura‐2 and EGFP. Because Fura‐2 and EGFP have overlapping excitation spectra at 380 nm but not at 340 nm, Fura‐2 signals excited at 340 nm are dominated by Fura‐2 fluorescence, whereas fluorescence excited at 380 nm reflects the sum of Fura‐2 and EGFP. This confound yields lower *F*_340_:*F*_380_ ratios in cells with strong EGFP expression compared with non‐EGFP cell types (Bolsover et al., 2001). To explore this possibility, we directly compared the changes of the fluorescence intensity excited at 340 nm in Merkel cells and non‐Merkel cells without calculating the *F*_340_:*F*_380_ ratios. Surprisingly, the mean peak fluorescence change (∆*F*_340_/*F*_340_) recorded in non‐Merkel cells (median: 0.11, *n* = 25) was still significantly stronger than Merkel cells by a factor of 2–3 (median: 0.05, *n* = 25, *p* < 0.01, two‐tailed Mann–Whitney test), suggesting that the calcium increases in the responding non‐Merkel epithelial cells were indeed much larger than those in Merkel cells.

It is unknown whether these non‐Merkel cells were directly activated by mechanical stimulation or indirectly activated through coupling with Merkel cells. To determine whether Merkel cells are required for the initiation of non‐Merkel cell responses, oral epithelia from mice that completely lack Merkel cells (*Atoh1* ^*CKO*^) were labeled with Fura‐2 and tested with mechanical stimulation. Mechanical stimuli induced calcium responses in 10/11 oral epithelial samples tested, demonstrating that Merkel cells are not required for mechanically evoked calcium responses in non‐Merkel epithelial cells (Figure S3a). Although *Atoh1* ^*CreERT2*^ *;Rosa26* ^*GCaMP6f*^ mice were not suitable for specifically measuring Merkel cell activity, GCaMP6f expressing outside Merkel cells in epithelia provided a potential tool to measure non‐Merkel cell activity. We discovered that following mechanical compression, the irregular GCaMP6f+ regions displayed clear responses in four of six epithelial tissues from four mice. These regions did not show individual Merkel cell morphology, strongly suggesting that they reflected the activation of some non‐Merkel cells in epithelia (Figure S3b). Together, these results demonstrate that oral epithelia harbor at least two types of epithelial cells capable of rapid activation by mechanical stimulation.

### 3.4 Functional significance of oral Merkel cells

**FIGURE 7:**
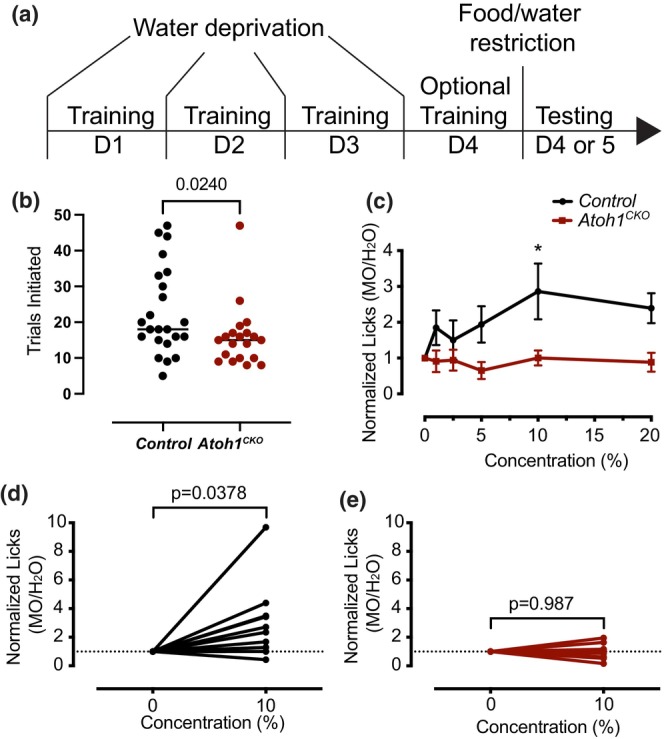
Merkel cells contribute to oily flavor preference in males. (a) Diagram depicting experimental paradigm. Mice were first trained for 3–4 days on drinking from a brief access window under water‐deprived conditions. After learning the task, mice were food and water restricted overnight, and then tested on preference for mineral oil emulsion (0%–20%). (b) *Atoh1* ^*CKO*^ mice initiated fewer trials upon testing than *Control* littermates (Mann–Whitney test, line denotes median). (c) Male mice have a moderate but significant preference for mineral oil emulsion (MO) that is lost in *Atoh1* ^*CKO*^ mice (Two‐Way Repeated Measures ANOVA *p*_concentration_ = 0.0416, *p*_genotype_ = 0.06, *p*_genotype × concentration_ = 0.0345, Holm‐Sidak's multiple comparisons *p < 0.05). (d) Control males consistently elicited a preference for 10% mineral oil emulsion (paired two‐tailed *t* test *p* = 0.0378). (e) *Atoh1* ^*CKO*^ male mice had no preference for 10% mineral oil compared to water (paired two‐tailed *t* test *p* = 0.9872). The number of licks is normalized to water.

To explore the potential role of food texture distinction for oral cavity Merkel cells in mammals, we tested a hypothesis that oral Merkel cells contribute to flavor preferences. In particular, we posited that Merkel cells play a role in the detection of mouth coating and viscosity sensations elicited by a creamy emulsion, which are orosensory texture cues for caloric fatty substances (Drewnowski & Almiron‐Roig, 2010). We tested mineral oil emulsion as it is nonnutritive and believed to be tasteless. In pilot studies, we tested whether *Atoh1* ^*CKO*^ affected preference for mineral oil emulsion in a two‐bottle preference test. We found that overall, mice did not elicit a strong preference for mineral oil in the two‐bottle test, thus we switched to brief access testing (Figure 7a). We tested short‐term preferences for mineral oil emulsions (0%–20%) using a gustometer. Overall, *Atoh1* ^*CKO*^ mice completed significantly fewer trials than *Control* mice (15.9 ± 8.7 vs. 22.9 ± 12.2, Mann–Whitney test, *p* = 0.024, *n* = 20–23 mice/group, Figure 7b). These data suggest that *Atoh1* ^*CKO*^ mice are not as motivated to receive mineral oil stimuli compared with *Control* mice. This effect was particularly pronounced in female *Atoh1* ^*CKO*^ mice, 7/12 of which were removed from further analysis for failing to complete 12 trials compared to 3/10 of *Control* females.

We further analyzed the preference for mineral oil emulsion in male mice that completed at least 12 trials. Males displayed a significant interaction between concentration and genotype, a significant effect of concentration, and a trend toward a significant effect of genotype (Figure 7c, *p*_concentration_ = 0.0416, *p*_genotype_ = 0.06, *p*_genotype × concentration_ = 0.0345, two‐way repeated measures ANOVA with Holm‐Sidak's post‐hoc test). Importantly, *Atoh1* ^*CKO*^ males had significantly fewer licks for 10% mineral oil by post‐hoc testing (*p* = 0.0266, Holm‐Sidak's post‐hoc test) and a trend toward fewer licks at 20% (*p* = 0.0968, Holm‐Sidak's post‐hoc test). We further analyzed the preference for 10% mineral oil emulsion (Figure 7d,e). We found that individual control animals had a consistent preference for 10% mineral oil compared to water (paired two‐tailed *t* test *p* = 0.0378). On the other hand, *Atoh1* ^*CKO*^ male mice showed no significant change in licks for 10% mineral oil compared to water (*p* = 0.987, paired two‐tailed *t* test). Collectively, these data suggest that *Atoh1* ^*CKO*^ animals have a modest loss of preference for or detection of mineral oil emulsion in brief‐access tests.

## 4 DISCUSSION

This study has established an ex vivo oral mucosa epithelial preparation for Merkel cell physiology studies and demonstrates that Merkel cells in the oral cavity are functional and activated by mechanical stimulation. Furthermore, we discovered that Merkel cells are critical in establishing preference for oil emulsion in mice and may play a role in food texture distinction.

### 4.1 Merkel cell physiology in ex vivo preparation for oral mucosa epithelia

Merkel cells that reside in the skin are activated by mechanical stimulation through nonselective cation Piezo2 channel activation, which subsequently activates high voltage‐activated calcium channel HVACC Cav2.1 and internal calcium stores (Maksimovic et al., 2014; Piskorowski et al., 2008; Woo et al., 2014). The action potentials generated in Merkel cells are mediated by voltage‐gated calcium channels while voltage‐gated sodium plays very little or no role (Ikeda et al., 2014). The calcium signals detected by GCaMP6f are a combination of calcium influx through Piezo2 channel, HVACC, and release from internal calcium stores.

The experimental configuration used in this study mimicked that of in vivo conditions, where mechanical forces were directly applied against the stratum corneum of gingiva or hard palate. Intracellular calcium increases in response to mechanical forces were observed in ~20% *VGLUT3* ^Cre^+ Merkel cells tested. Although the native pressure on the gingiva or palate in mice is unknown, our data suggest that the pressure required to activate the Merkel cells in the mouse hard palate (corresponding to 8%–40% strain, 5–75 kPa) is comparable with that human hard palate faces during oral processing and swallowing (3–40 kPa) (Fujiu‐Kurachi et al., 2014; Yokoyama et al., 2014). These findings indicate that our ex vivo live‐cell imaging of the oral mucosa epithelial preparations is an accurate approximation of in vivo conditions in mammals.

Merkel cells in oral mucosa can be activated as quickly as our system could stably image (0.7–1.3 s) following mechanical stimulation, with 50% of responses initiating within 1.3 s (Figure S2a), supporting the hypothesis that oral Merkel cells are mechanoreceptors. These Merkel cells could contribute to mechanosensory control of food intake. Interestingly, Merkel cells did not respond uniformly to mechanical stimuli, displaying a range of temporal patterns and mechanical thresholds. We found that Merkel cells in gingiva had a lower mean mechanical threshold than those in the hard palate, implying that Merkel cells in the gingiva are more mechanically sensitive than in the hard palate. Although direct comparisons between murine gingiva and palate mechanical threshold have not been reported to our knowledge, the human gingiva has a lower pressure‐pain‐threshold than palate suggesting that gingiva is more mechanosensitive than hard palate (Ogawa et al., 2004; Ogimoto et al., 2002). It is possible that factors, such as the hardness of supporting tissues (bone, tooth, lamina propria), may contribute to the mechanical sensitivity of gingival and palatal Merkel cells in vivo. These factors were not accounted for in this ex vivo preparation and thus may not completely recapitulate mechanical forces found in vivo. This difference in threshold identified in this study is likely due to the difference in Merkel cell sensitivity, not epithelial tissue mechanics as gingiva and hard palate showed similar mechanical properties. Our earlier studies found that individual isolated Merkel cells from neonatal skin exhibit variable current–displacement relationships and touch‐evoked ionic current time courses (Maksimovic et al., 2014), supporting the idea that Merkel cells in different tissues may show different sensitivity to mechanical stimulation. It is unclear whether this disparity in sensitivity was due to variations in morphology, molecular identities, or the spatial distribution of Piezo channels as Merkel cells are polymorphic in the oral mucosa (Tachibana et al., 1997), our data support the idea that differences in Piezo2 expression or distribution in Merkel cells may contribute to the different mechanical thresholds.

Based on the calcium imaging data from *VGLUT3* ^*Cre*^ *; Rosa26* ^*(GCaMP6f/tdTomato)*^ and J2XnGFP mice, we estimated the percentage of Merkel cells activated following mechanical stimulation. It is interesting that only ~20% of the Merkel cells were activated in this experimental configuration, raising the possibility that some Merkel cells could not be activated due to the damage during epithelium preparation or deleterious effects of high tdTomato expression. These are not likely to be the case as unhealthy cells are usually accompanied by higher internal calcium, a condition that was not observed in Fura‐2 experiments. In addition, virtually all Merkel cells were activated with high K+ superfusion, demonstrating that the majority of Merkel cells were activatable under these experimental conditions. One possibility for the discrepancy between mechanically activated Merkel cells compared with Merkel cells that respond to high K+ is that some could not be equipped with the full complement of machinery required for mechanical activation despite expressing enough voltage‐gated calcium channels to be activated by membrane depolarization. Another possible explanation for the low percentage of activated Merkel cells following mechanical stimulation is that stronger stress is required to stimulate some Merkel cells or that transduction of mechanical forces is incomplete without supporting tissues. We do not exclude the possibility that some Merkel cells serve roles other than sensory signaling (Eispert et al., 2009). Further studies are required to uncover their low activation rate and whether this has a physiological significance.

### 4.2 Identification of Merkel cells with markers

This investigation has also revealed a previously unreported population of *VGLUT3*‐lineage epidermal cells. In our study, all hard palatal and gingival clustered *VGLUT3*‐lineage cells expressed K8. However, very few scattered *VGLUT3*‐lineage cells in gingiva expressed the Merkel‐cell marker K8. Interestingly, these *VGLUT3*‐lineage/K8‐negative cells also responded to mechanical stimulation, suggesting a special group of cells that were not typical Merkel cells may also play a role in detecting mechanical stimulation. These data suggest that VGLUT3 is a good Merkel‐cell marker in the oral cavity, although it also labels some unidentified cell types in gingiva. Most importantly, 91% of the K8+ cells in the hard palate and 98% of the clustered K8+ cells in the gingiva expressed VGLUT3^Cre^, demonstrating that most mature typical Merkel cells in the oral mucosa were of *VGLUT3* lineage.

An earlier study showed that ≥90% of Atoh1‐expressing epidermal cells are K8+ Merkel cells in hairy skin and whisker follicles following three tamoxifen injections (250 mg/kg) to activate Cre recombinase (Wright et al., 2015). Because our trials showed strong GCaMP6f expression in *Atoh1* ^*CreERT2*^ *;Rosa26* ^*GCaMP6f*^ mice and many were K8‐negative cells, we reduced the amount and number of tamoxifen injections (100 mg/kg, one injection). Still, in our study, 80%–95% of the GCaMP6f+ region in hard palate and gingiva did not show K8 immunoreactivity. It is unclear why many Atoh1‐lineage cells in the oral epithelium were not Merkel cells as Atoh1 expression is sufficient to drive K8 expression throughout the epidermis (Ostrowski et al., 2015). One possible explanation is that these *Atoh1*‐lineage GCaMP6f + cells in *Atoh1* ^*CreERT2*^ *;Rosa26* ^*GCaMP6f*^ oral mucosa might be squamous derivatives of a bipotential Merkel‐cell progenitor from the *Atoh1* lineage, located in epithelium (Morrison et al., 2009; Woo et al., 2010). In support of this, we find many GCaMP6f+ cells located at the stratum spinosum of the epithelia, especially those irregularly shaped cells. Interestingly, these GCaMP6f cells also responded to mechanical stimulation and showed calcium responses (Figure S3b).

### 4.3 Mechanosensitivity of Merkel cells and non‐Merkel cells in oral mucosa

Based on fura‐2 calcium imaging experiments on J2XnGFP mice and GCaMP6f experiments using *Atoh1* ^*CreERT2*^ *;Rosa26* ^*GCaMP6f*^ mice, non‐Merkel epithelial cell types also responded to mechanical stimulation, indicating that other mechanosensitive cells may contribute to stress sensing in oral mucosa. This is particularly interesting because these non‐Merkel cells showed stronger calcium responses than typical Merkel cells. These non‐Merkel cell responses were also observed in *Atoh1* ^*CKO*^ mice, demonstrating that Merkel cells are not required for activation. In addition, scattered GCaMP6f+/K8‐cells in gingival epithelia and irregular GCaMP6f+ cells in *Atoh1* ^*CreERT2*^ *;Rosa26* ^*GCaMP6f*^ epithelia can be activated by mechanical stimulation, strongly arguing that Merkel cells and non‐Merkel cells in the oral mucosa can be independently activated and may play distinct roles in transmitting sensory information to the nervous system or cellular homeostasis. These mechanosensitive non‐Merkel cells are likely to be keratinocytes due to their abundance and as calcium waves were observed in the preparation (Tsutsumi et al., 2009). The role of the mechanosensitivity of the keratinocytes in oral mucosa remains to be determined. Nevertheless, some epidermal keratinocytes have been suggested to mediate sensory modalities, including pain and tactile sensation (Baumbauer et al., 2015; Pang et al., 2015). Also, increased cytoplasmic‐free calcium may play a critical role in mediating the fate of epithelial cells as the type of mechanical force controls the outcome: stretch induces cell division, whereas crowding induces extrusion (Gudipaty et al., 2017). Although isolated Merkel cells respond to mechanical stimulation and epithelial cell activation is not required for their activation (Higashikawa et al., 2019; Maksimovic et al., 2014), we do not exclude the possibility that activated keratinocytes may also modulate Merkel cell activity in the epithelia. Future studies with keratinocyte‐specific genetic drivers may help to further dissect the biological basis of mechanosensitivity in these mechanosensitive non‐Merkel cells in oral mucosa, and to define whether Piezo2 is responsible for their mechanosensitivity.

### 4.4 Functions of oral Merkel cells in distinguishing oil emulsions

Our findings that oral Merkel cells are abundant in oral tissues and mechanosensitive raise the question as to how they contribute to flavor sensation. We surmised that as Merkel cells encode sustained pressure (Maksimovic et al., 2014; Nakatani et al., 2015), they may contribute to either sensation of mouth‐coating of oil emulsions or to viscosity perception through the detection of pressure on the hard palate due to shear‐thinning during consumption (Deblais et al., 2021). We developed assays to test preference for mineral‐oil emulsions. We found that overall, mice only modestly prefer mineral oil under these testing conditions. Regardless, we found significantly decreased preference for emulsion in *Atoh1* ^*CKO*^ with gustometer testing. Interestingly, *Atoh1* ^*CKO*^ mice initiated significantly fewer trials in mineral oil gustometer testing, indicating that there may be a deficit in detecting mineral oil. Collectively, this provides the first evidence that oral Merkel cells contribute to flavor preference. Future studies should assess whether Merkel cells are necessary for the detection of oils or other flavor compounds associated with mechanosensation.

In summary, we have successfully established a system to assay intact ex vivo epithelial physiology and conducted the first functional study of Merkel cells in live oral mucosa tissues. We also demonstrated that both Merkel cells and non‐Merkel epithelial cells responded to mechanical stimulation. Finally, we found that Merkel cells contribute to preference for oily emulsion. This study not only provides the foundation for future investigation of Merkel and non‐Merkel cell physiology and their relation with oral texture sensation, but also suggesting a new role of Piezo2 channel in mediating food texture sensation in mammals.

## AUTHOR CONTRIBUTIONS

*Conceptualization*: Ellen A. Lumpkin, Chi‐Kun Tong, Yalda Moayedi. *Methodology*: Ellen A. Lumpkin, Yalda Moayedi, Chi‐Kun Tong. *Programming*: Chi‐Kun Tong, Yalda Moayedi. *Validation*: Ellen A. Lumpkin, Chi‐Kun Tong. *Formal analysis*: Chi‐Kun Tong, Yalda Moayedi. *Investigation*: Ellen A. Lumpkin, Yalda Moayedi, CKT. *Resources*: Ellen A. Lumpkin, Yalda Moayedi, Chi‐Kun Tong. *Data curation*: Chi‐Kun Tong, Yalda Moayedi. *Writing—Original draft preparation*: Chi‐Kun Tong. *Writing—Review and editing*: Yalda Moayedi, Ellen A. Lumpkin. *Visualization*: Chi‐Kun Tong, Yalda Moayedi, Ellen A. Lumpkin. *Supervision*: Ellen A. Lumpkin. *Project administration*: Ellen A. Lumpkin. *Funding acquisition*: Ellen A. Lumpkin.

## FUNDING INFORMATION

This work was supported by SPN, formerly NESTEC S.A., NIAMS R01AR051219, Epicure Grant #P30AR069632 for microscopy.

## CONFLICT OF INTEREST STATEMENT

The authors declare no conflict of interest related to this research.

## Supporting information


Figure S1.

Figure S2.

Figure S3.

## Data Availability

All study data are included in the article and/or Supporting Information.
